# Cost-effectiveness analysis of sintilimab plus pemetrexed and platinum versus chemotherapy alone as first-line treatment in metastatic non-squamous non–small cell lung cancer in China

**DOI:** 10.1186/s13561-022-00410-x

**Published:** 2022-12-30

**Authors:** Huiqin Liu, Ying Wang, Qi He

**Affiliations:** 1 Department of Medical Insurance, Cancer Prevention and Treatment Institute of Chengdu, Chengdu Fifth People’s Hospital (The Second Clinical Medical College, Affiliated Fifth People’s Hospital of Chengdu University of Traditional Chinese Medicine), Chengdu, China; 2 Department of Oncology, Cancer Prevention and Treatment Institute of Chengdu, Chengdu Fifth People’s Hospital (The Second Clinical Medical College, Affiliated Fifth People’s Hospital of Chengdu University of Traditional Chinese Medicine), Chengdu, China

**Keywords:** Non-small cell lung cancer, Sintilimab, Cost-effectiveness analysis, Partitioned survival model

## Abstract

**Objective:**

In frst-line treatment of advanced or metastatic nonsquamous non-small-cell lung cancer (NSCLC), the ORIENT-11 study demonstrated a signifcant progression-free survival and overall survival for sintilimab plus chemotherapy in comparison with chemotherapy alone. But the cost-effectiveness of the two treatment schemes is unclear in China. The objective of the current study was to evaluate the cost efectiveness of sintilimab plus chemotherapy versus Platinum-based chemotherapy for locally advanced or metastatic squamous NSCLC in China.

**Methods:**

We performed an economic evaluation from the perspective of the Chinese healthcare system using a partitioned survival model with three mutually exclusive health states: progression free, post-progression, and death. The circulation cycle of the model was 3 weeks and the study time limit was 10 years. Efficacy data were obtained from the ORIENT-11 clinical trial. Cost and utility values were derived from published studies and online price databases. The primary outcomes of the model were quality-adjusted life-years (QALYs), costs, and incremental cost-effectiveness ratios (ICERs). One-way sensitivity analysis and probability sensitivity analysis were used to verify the robustness of the base-case analysis results.

**Results:**

Sintilimab plus chemotherapy provided an additional 0.6 QALYs. The total cost per patient was CNY¥413,273.16 for sintilimab plus chemotherapy and CNY¥280,695.23 for Platinum-based chemotherapy. The ICER for sintilimab plus chemotherapy was CNY¥220,963.22/QALY. Sensitivity analyses found the results to be most sensitive to the cost of pemetrexed and utilities of PF state. In the probabilistic sensitivity analysis, sintilimab was cost-efective in 78.6% of the simulations, assuming a willingness-to-pay threshold (WTP) of CNY¥242,928 per QALY.

**Conclusion:**

Compared with chemotherapy alone, the sintilimab plus chemotherapy is likely to be a cost-effective option as the first-line treatment for locally advanced or metastatic nonsquamous NSCLC in China.

## Background

Lung cancer is one of the most commonly diagnosed cancers worldwide. In 2020, there were about 19,292,789 new cancer cases worldwide, with a crude incidence rate of 247.5/100,000 and ASIRW of 201.0/100,000. Among them, there were 2,206,771 new cases of lung cancer and 1,796,144 deaths, accounting for 11.4% and 18.0% of the total new cancer cases and deaths, respectively. In China, the number of new cases and deaths of lung cancer ranked first in 2020. In 2020, there were 3million cancer deaths in China, and 710,000 lung cancer deaths, accounting for 23.8% of cancer deaths [1]. Non-small cell lung cancer (NSCLC) accounts for 80%-85% of lung cancers, mainly occurring in the bronchial mucosa, bronchial glands and alveolar epithelium, and 70% of patients are diagnosed at an advanced stage.

NSCLC imposes a significant burden to patients and health care systems in China. According to the data from the China Health Statistics Yearbook 2021 [2], the average hospitalization cost of lung cancer inpatients in China was ¥23,871.48, the average hospital stay was 11.6. days in 2020. Sintilimab is a selective anti-PD-1 antibody that inhibits interactions between PD-1 and its ligand, PD-L1. The recent phase III ORIENT-11 trial showed that in patients with EGFR and ALK mutation-negative advanced or metastatic nonsquamous NSCLC, the addition of sintilimab to standard chemotherapy with pemetrexed and platinum, significantly prolonged PFS compared (8.9 mo versus 5.0 mo, HR, 0.482, *p* < 0.00001) [3]. Given the new efficacy evidence for sintilimab, there are two possible treatment strategies available for this patient population: standard chemotherapy using pemetrexed and first-line use of sintilimab. Comparative cost-effectiveness evidence between these treatments is required to inform coverage decision making. In addition, sintilimab plus chemotherapy was included in the National Medical Insurance Drug List in the 2021 China Medical Insurance Negotiations in December 2021. And it has prompted the second drop of the sintilimab price since sintilimab entered the medical insurance catalog in 2020, with a decrease of 62.0%. Therefore, our objective was to compare cost-effectiveness of these two treatment strategies for patients with advanced NSCLC without sensitizing EGFR or ALK mutations.

## Materials and methods

### Patient material and clinical data

This study was based on a randomized, double-blind, placebo-controlled phase 3 clinical trial study (ORIENT-11). In ORIENT-11, a total of 397 eligible patients were randomized (2:1 ratio) to receive either sintilimab 200 mg or placebo plus pemetrexed and platinum once every 3 weeks for four cycles, followed by sintilimab or placebo plus pemetrexed therapy. Eligible patients were aged 18 to 75 years old with histologically or cytologically confirmed stage IIIB to IV nonsquamous NSCLC who were ineligible for radical surgery or radiotherapy, had no sensitive EGFR mutations or anaplastic lymphoma kinase rearrangements. Crossover or treatment beyond disease progression was allowed.

### Model structure

A partitioned survival model (PSM) was developed to estimate the costs and outcomes of patients from a Chinese healthcare system perspective. According to the development of NSCLC, the disease was divided into three health states: Progressed Free Survival (PFS), Post Progression(PP), and Death [4]. We assumed that all patients in the model entered the model in the PF health state. The cycle length was set as 3 weeks (21 days), which was aligned with the administration cycle of the drugs in the ORIENT-11, and the time horizon was 10 years.The main outcomes of the model output were total cost, quality-adjusted life-years (QALYs), and incremental cost-effectiveness ratio (ICER). According to the *China Guidelines for Pharmacoeconomic Evaluations 2020*, all costs and health outcomes were calculated based on a discount rate of 5%, and 3 times GDP per capita in China was considered as a willingness-to-pay threshold for the cost-effective analysis. It was assumed that costs were incurred at the beginning of each treatment cycle, so no half-cycle correction for costs were required, and health outcome data were subjected to half-cycle correction.

The survival analysis of this study was completed in R4.1.1, and the Partitioned Survival Model (PSM) and cost-effectiveness analysis were completed in Microsoft Excel 2019.

### Survival analysis

**Fig. 1:**
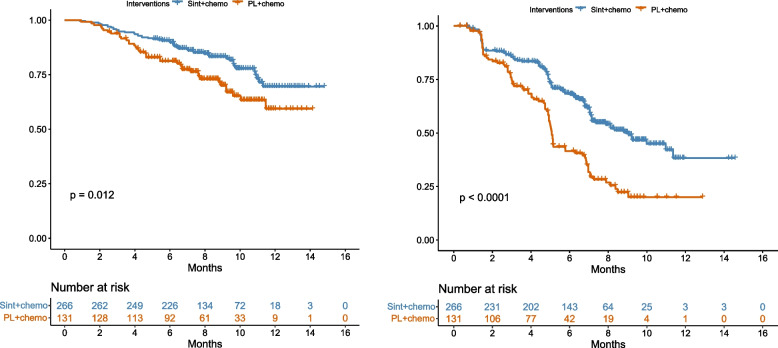
Reconstructed OS curve and PFS curve

**Fig. 2:**
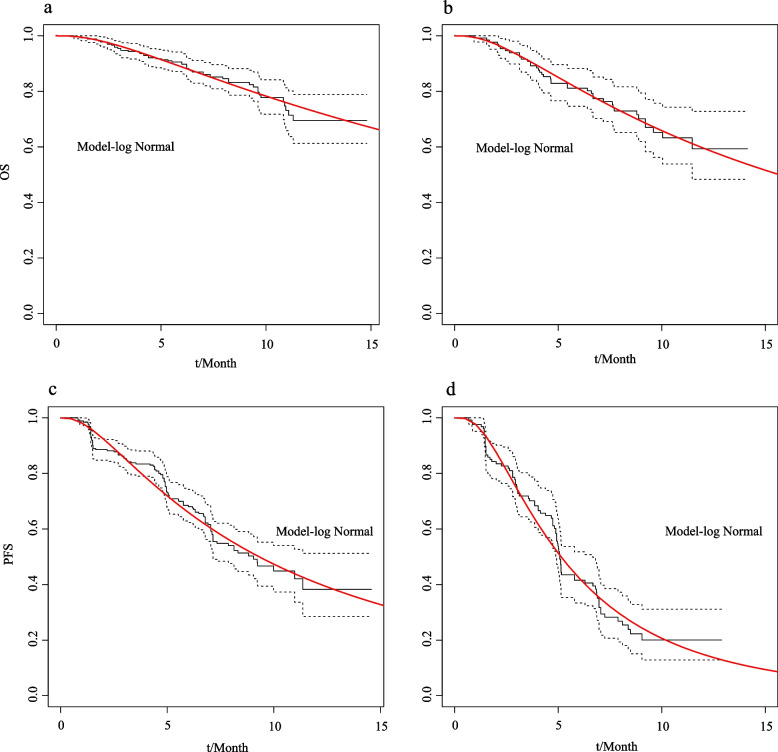
OS curve and PFS curve fitting in the experimental group and control group (**a, c**-experimental group; **b, d**-control group)

The proportion of patients in different health states during the trialperiod was directly obtained from the OS and PFS curves of the ORIENT-11 study using GetDataGraphDigitizer software. The proportion of patients in progression-free state was directly provided by the PFS curve, and the proportion of patients in death state was obtained from (1-overall survival rate), the proportion of persons in progressive status was the difference between the OS and PFS curve survival rates. Survival data beyond the follow-up period were extrapolated by survival curve fitting. Firstly, use the GetDataGraphDigitizer software to extract the data points from the OS and PFS Kaplan–Meier curves of the experimental group and control group respectively. Based on the OS and PFS data, the survHE package of R(V4.1.1) was used to fit and extrapolate the PFS and OS curves [5],  and the reconstructed OS/PFS curve and exploration and fitting of OS/PFS are shown in Figs. 1 and 2. Maximum Likelihood Estimation (mle) and Hamiltonian Monte Carlo (HMC) were adopted to parameter estimate respectively. Secondly, according to the values of Akaike information criterion (AIC), Bayesian Information Criterion (BIC) and Deviance Information Criterion (DIC) combined with visual inspection, the survival parameter values of the optimum fitting distribution were obtained. The AIC, BIC and DIC values of different KM curve fitting distributions are shown in Tables 1 and 2, and the optimum fitting distribution and distribution parameters of different KM curves are shown in Table 3.

**Table 1:** AIC and BIC of OS curve and PFS curve in the Sintilimab group and chemotherapy group (mle)

group	exponential		gamma		genf		gengamma	
	AIC	BIC	AIC	BIC	AIC	BIC	AIC	BIC
OS curve(sintilimab-combination group)	496.88	500.46	487.56	494.73	491.22	505.55	489.22	499.97
OS curve(placebo-combination group)	333.26	336.14	328.12	333.87	329.21	340.71	327.21	335.84
PFS curve(sintilimab-combination group)	823.26	826.84	807.58	814.74	809.64	823.97	807.64	818.39
PFS curve(placebo-combination group)	513.02	515.89	489.20	494.95	491.40	502.90	489.40	498.03
group	weibull		weibullPH		loglogistic		lognormal	
	AIC	BIC	AIC	BIC	AIC	BIC	AIC	BIC
OS curve(sintilimab-combination group)	487.83	494.99	487.83	494.99	487.73	494.90	487.30	494.47
OS curve(placebo-combination group)	328.95	334.70	328.95	334.70	327.80	333.55	325.84	331.59
PFS curve(sintilimab-combination group)	808.83	816.00	808.83	816.00	807.72	814.89	805.67	812.84
PFS curve(placebo-combination group)	491.88	497.63	491.88	497.63	488.61	494.36	487.54	493.29

**Table 2:** AIC BIC and DIC of OS curve and PFS curve in the Sintilimab group and chemotherapy group (HMC)

group	exponential			gamma			genf			gengamma		
	AIC	BIC	DIC	AIC	BIC	DIC	AIC	BIC	DIC	AIC	BIC	DIC
OS curve(sintilimab-combination group)	498.88	506.05	496.86	489.63	500.38	487.39	493.82	511.74	489.17	492.81	507.14	480.77
OS curve(placebo-combination group)	335.27	341.02	333.32	330.15	338.77	328.25	331.91	346.28	327.53	329.40	340.90	327.08
PFS curve(sintilimab-combination group)	825.26	832.42	823.31	809.58	820.33	807.48	813.72	831.63	809.41	809.66	823.99	807.76
PFS curve(placebo-combination group)	515.02	520.77	512.99	491.22	499.85	489.31	494.54	508.92	490.56	491.43	502.93	489.34
group	weibull			weibullPH			loglogistic			lognormal		
	AIC	BIC	DIC	AIC	BIC	DIC	AIC	BIC	DIC	AIC	BIC	DIC
OS curve(sintilimab-combination group)	489.86	500.61	487.96	489.86	500.61	487.76	489.77	500.52	487.92	489.43	500.18	487.35
OS curve(placebo-combination group)	331.00	339.63	329.10	370.16	378.79	751.00	329.83	338.46	327.99	327.90	336.52	325.86
PFS curve(sintilimab-combination group)	810.84	821.59	808.83	810.83	821.58	808.76	809.74	820.49	807.58	807.72	818.47	805.72
PFS curve(placebo-combination group)	493.89	502.52	491.92	493.90	502.52	491.83	490.62	499.25	489.02	489.60	498.23	487.54

**Table 3:** KM curve optimum fitting distribution and parameters

	Optimum fitting distribution	Survival function	Fitting parameters	
			μ	σ
OS curve(sintilimab-combination group)	lognormal	S(t) = 1-φ〔(logt-μ)/σ〕	3.2277	1.1832
OS curve(placebo-combination group)	lognormal		2.7519	1.1003
PFS curve(sintilimab-combination group)	lognormal		2.2309	1.0708
PFS curve(placebo-combination group)	lognormal		1.6359	0.8141

### Treatment costs

This analysis adopted a health care perspective in China, only direct medical cost was calculated, which included patient drug costs, drug management costs, disease management costs, adverse events (AEs) costs.

### Drug cost

Drug costs included the cost of sintilimab, pemetrexed, chemotherapy drugs, and subsequent treatments. After disease progression, patients in the control group were crossed over to receive sintilimab monotherapy. ORIENT-11 did not provide detailed instructions on the drug use after progression in the trial group, according to the *third edition of the non-small cell lung cancer guidelines* published by America National Comprehensive Cancer Network(NCCN) in 2021, it was assumed that after disease progression in the experimental group, immune checkpoint inhibitors were no longer used and chemotherapy regimens were adopted, that is, docetaxel was selected for follow-up treatment, and patients who had not progressed after 24 months of maintenance treatment were treated with the best supportive care, see Table 4. The drug costs of treatment were based on the median price of the winning bid product derived from the China Drug Bidding Database (shuju.menet.com.cn) and published articles [6–8]. To estimate the dosages of pemetrexed in the sintilimab arm and the placebo arm, we assumed that the mean body surface area was 1.74 m2.

**Table 4:** Basic values and variation ranges of model parameters

Parameter	Baseline value(¥)	Usage and dosage	Range(DSA)	Distribution(PSA)	Reference
Sintilimab	2160/200 mg	200 mg/3 week	5686–1512	log-normal	MENET
Carboplatin	1.12/mg	511.87 mg/3 week	± 30%	log-normal	[6]
Cisplatin	1.35/mg	130.5 mg/3 week	± 30%	log-normal	[7]
Pemetrexed	2142/100 mg	870 mg/3 week	± 30%	log-normal	[6]
Docetaxel	1300/200 mg	104.4 mg/3 week	± 30%	log-normal	[8]
Best supportive care	2336		± 30%	log-normal	[6]
Drug management costs(sintilimab-combination group)	56.25		± 30%	log-normal	[8]
Drug management costs(placebo-combination group)	315.25		± 30%	log-normal	[6]
Disease management costs(sintilimab-combination group)	372.42		± 30%	log-normal	[6]
Disease management costs(placebo-combination group)	401.42		± 30%	log-normal	[6, 9]
Adverse event					
Anemia	1.17	S: 15.0% P: 19.1%			[6]
Decreased neutrophil count	2877.4	S: 36.5% P: 30.5%			[10]
Decreased white blood count	7845	S: 14.7% P: 15.3%			[6]
Decreased platelet	23,086.04	S: 12.0% P: 12.2%			[11]
Cost of managing adverse event(sintilimab-combination group)	4973.97		± 30%		
Cost of managing adverse event(placebo-combination group)	4894.61		± 30%		
Effectiveness					
PF state	0.815		15%	Beta	[12]
PD state	0.321		15%	Beta	[12]
Death state	0				
Discount rate	5%		3%-8%

**Table 5:** Summary of the cost and health outcomes results

	Sintilimab plus chemotherapy	Placebo plus chemotherapy
QALYs	1.52	0.92
PF QALYs	1.00	0.47
PP QALYs	0.52	0.45
Total costs	¥413,273.16	¥280,695.23
Drug costs	¥368,860.32	¥200,158.20
Administration costs	¥2753.56	¥13,762.61
Disease	¥18,230.80	¥10,808.29
management and		
monitoring costs		
AE costs	¥4973.97	¥4894.61
Subsequent	¥18,454.51	¥51,071.52
therapy costs		
Average cost-effectiveness ratios (ACER)	¥271,890.23/QALY,	¥305,103.51/QALY
Incremental costs	¥132,577.93	
Incremental QALYs	0.60	
ICER	¥220,963.22/QALY	

### Adverse events

AEs with an incidence ≥ 5% and grade ≥ 3 in the ORIENT-11 studies were included in our study, including anemia, decreased white blood cell count, decreased platelet,and decreased neutrophil count. The total incidence of other AEs decreased to below 5% in the experimental group and below 10% in the control group. The incidence of AEs were derived from ORIENT-11. The costs related to AEs were calculated by multiplying the incidence of the serious AEs by the costs of managing the serious AEs per event. In clinical practice, physicians may make a decision to discontinue or change the drug if adverse events ≥ grade 3 occur. Therefore, the model assumed that the treatment of adverse events was a one-time cost.AEs costs were based on the data from previously published studies [6, 10, 11].

### Disease management costs and drug management costs

Drug management costs incurred by patients during treatment include diagnostic fees, intravenous injection fees, nursing fees and bed fees. Disease management costs include contrast-enhanced CT of the chest, blood routine, urine routine, and blood biochemistry.The unit costs of disease management and drug management were from the healthcare documents and expert opinion [6, 8, 9].

### Utility estimates

Utility was applied to measure patient’s preference for living at a particular health state, where 0 stood for the worst health and 1 for the best. It reflected the impact of the disease-related health states. We used utilities of 0.815 and 0.321, according to a health utility study in Chinese NSCLC patients by Nafees [12].

## Results

### Base case results

The result of the base-case analysis is presented in Table 5. In the base-case analysis, the average cost-effectiveness ratios (ACER) of patients in the Sintilimab group and placebo group were ¥271,890.23/QALY, ¥305,103.51/QALY, respectively. Patients in the Sintilimab group obtained an additional 0.6 QALYs, but needed to pay an extra ¥132,577.93. The incremental cost-effectiveness ratio was ¥220,963.22 /QALY.According to the guidelines of World Health Organization for cost-effective analysis, the threshold of willingness to pay (WTP) was evaluated at CNY¥242,928/QALY, three times of the Gross Domestic Product per Capita (GDP) of China in 2021. The ICER values were less than 3 times GDP per capita and the results demonstrate that Sintilimab plus chemotherapy is cost-effective compared with placebo plus chemotherapy.

### Deterministic sensitivity analysis

**Fig. 3:**
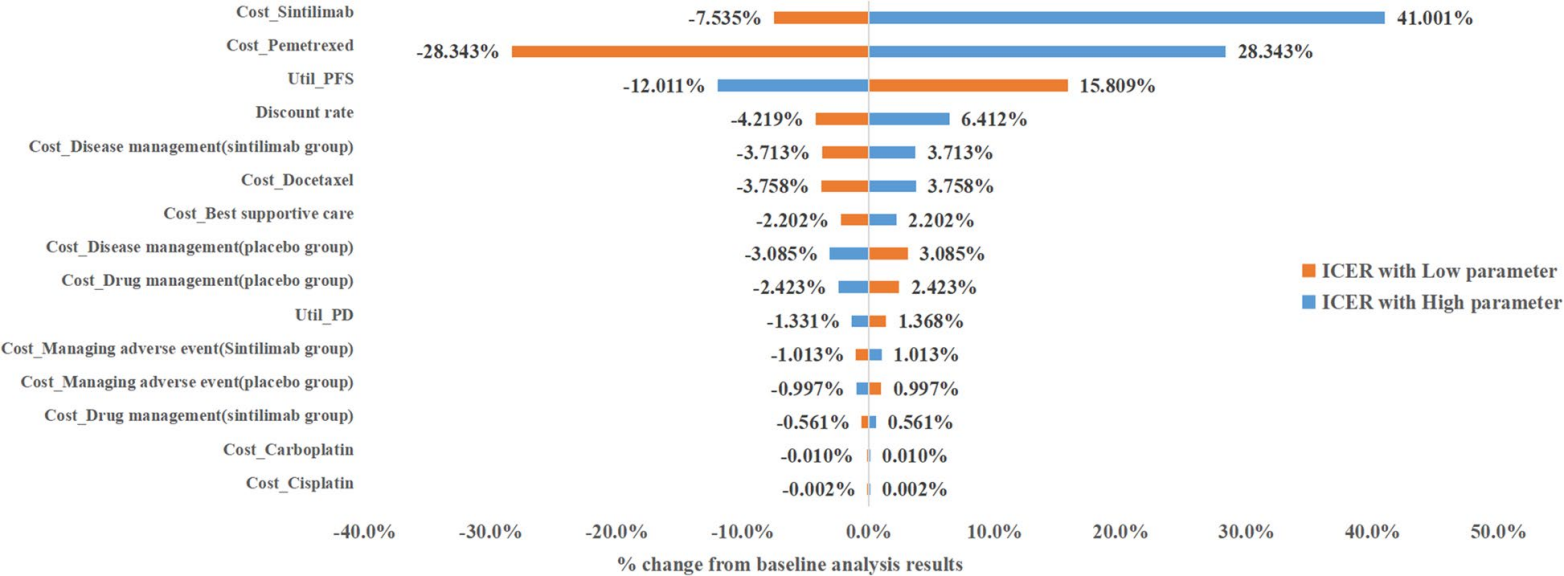
Deterministic sensitivity analysis of cost-effectiveness comparison of experimental group and control group

One-way sensitivity analyses were conducted to test the robustness of the base-case ICER, the results of which were shown as a tornado diagram to identify key factors (Fig. 3). A 30% change range was assumed for the parameters of drug price, medical service cost, and follow-up cost. A 15% change range was assumed for the parameters of PFS and PD utility. The base-case ICER was most sensitive (> 15% difference) to changes in the cost of Sintilimab, the cost of Pemetrexed, and utilities of PF state. The ICER was least sensitive to changes in the cost of Cisplatin and Carboplatin (≤ 0.1% difference).Under the price of sintilimab before the medical insurance price adjustment in January 2022 (¥5686), the cost-effectiveness analysis results have been economically reversed.

### Probabilistic sensitivity analysis

**Fig. 4:**
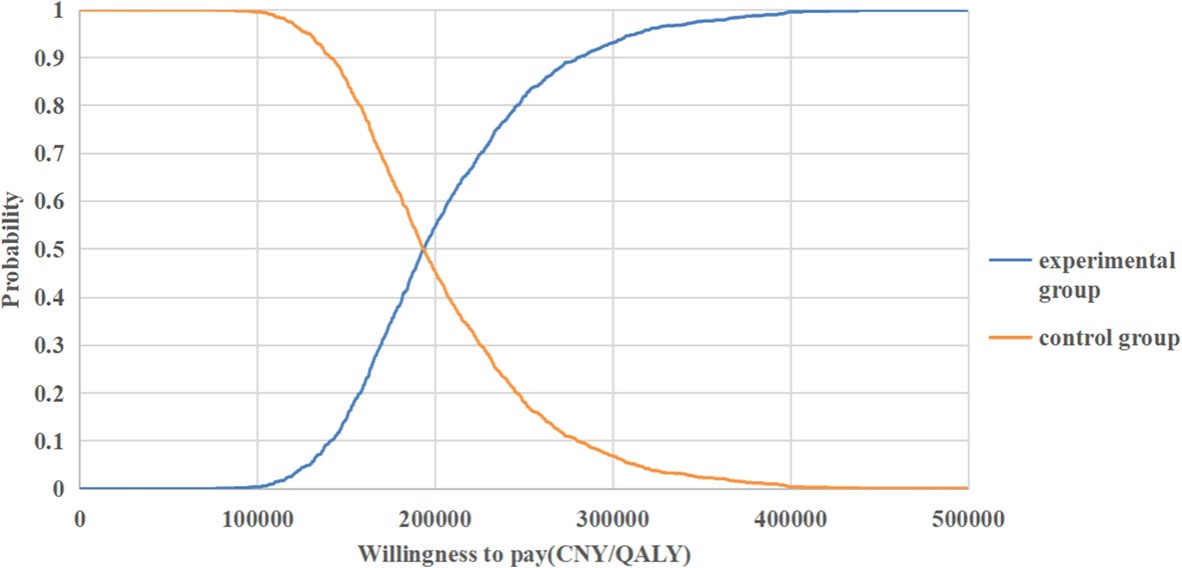
Cost-effectiveness acceptability curve

**Fig. 5:**
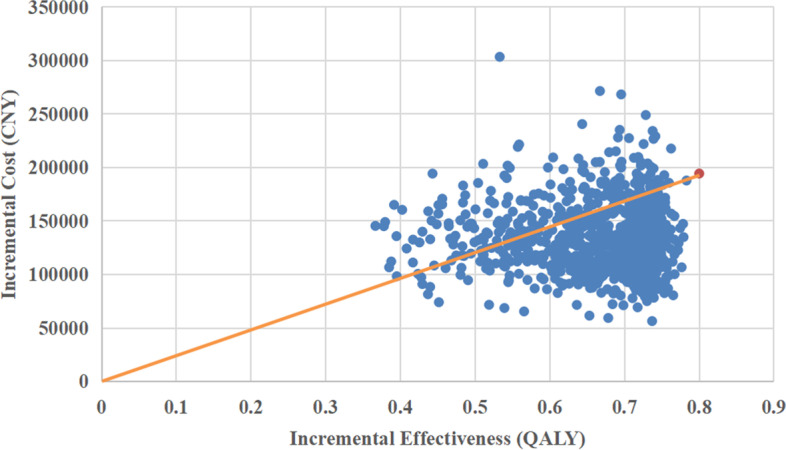
ICER scatterplot from probabilistic sensitivity analysis

The probabilistic sensitivity analysis was performed to estimate the probability of Sintilimab treatment being cost-effective compared to chemotherapy treatment. 1000 Monte Carlo simulations were conducted by inputting values drawn from their statistical distributions which were showed in Table 4. PSA results were presented using cost-effectiveness acceptability curves and the ICER scatterplot (Figs. 4 and 5) that show the Sintilimab to be cost-efective in 78.6% of the simulations, assuming a willingness-to-pay threshold (WTP) threshold of CNY¥242,928 per unit of QALY.

## Discussion

Sintilimab, as a selective anti-PD-1 antibody,by inhibiting the interaction between PD-1 and its ligand PD-L1, effectively relieve the immunosuppressive effect of the body, and enhance the activity of T cells, thereby greatly enhancing the killing ability and immune surveillance ability of T cells on tumors, and inhibiting the proliferation of tumor cells [13]. Some studies have evaluated the cost-effectiveness of immunotherapy in NSCLC from the perspective of payers in China [8, 14–18]. These published economic evaluations of advanced NSCLC in China mainly compared pembrolizumab or camrelizumab with chemotherapy and almost all studies have shown that immunotherapy is unlikely to be a cost-effective option compared to chemotherapy alone, although the ICERs reported by these studies vary. Recently, Sintilimab, a domestic PD-1 inhibitors, which has good cost performance, has provided more medication options for Chinese NSCLC patients. In addition, Sintilimab plus pemetrexed and platinum as the first-line treatment for patients with EGFR and ALK mutation-negative advanced or metastatic nonsquamous NSCLC was included in the National Medical Insurance Drug List in the China Medical Insurance Negotiations in December 2021. But there is a lack of economic evidence focused on domestic PD-1 inhibitors like Sintilimab. To the best of our knowledge, this is the first study to compare the cost-effectiveness of sintilimab plus chemotherapy vs chemotherapy for NSCLC.

The results showed that the significant price cuts of Sintilimab reducing the economic burden of patients. With the threshold of 3 times per capita GDP, sintilimab combined with chemotherapy drugs is cost-effective.However, the prices of combination drugs are still high, which also increases the medication cost for patients. The results of the sensitivity analysis indicated that the price of pemetrexed has a great impact on the ICER, and lowering its price will significantly reduce the ICER value, which suggests that companies should conduct more in-depth exploration of the monotherapy of immunotherapy drugs in the first-line treatment of lung cancer. In addition, in China where the coverage of social medical insurance system exceeds 95%, enter the medical insurance catalogue through price cuts may be an important pathway to gain more market share in the fierce competition,which could reduce the transaction costs for transnational pharmaceutical companies, such as finding transaction partners (hospital, etc.), promoting, establishing contract relationships, and fulfilling contracts. Meanwhile, administrative departments need to further improve the relevant health industry innovation policies such as how to create a free environment for the highly regulated medical and health industry and how to make the basic elements required for innovation have liquidity, to ameliorate the situation that China's innovative drugs and patented drugs rely heavily on imports [19]. And government also should adopt more aggressive health care regulatory policies to avoid wasting health care resources [20]. It is also suggested to accelerate the development of commercial health insurance by promoting Internet usage and increasing financial knowledge, and form a joint force with social medical insurance to meet the diversified health security needs of the masses [21, 22]. Moreover, in the ORIENT-11 trial, allowing patients in the control group to cross over to use sintilimab after disease progression, which have a positive effect on prolonging the patient's life, but also reduce the difference in QALY value between the trail group and the control group. And the economic advantage of sintilimab plus chemotherapy in the first-line treatment may be underestimated.

There are several limitations to this study. First, this study is inherently dependent on the validity and extrapolation of the clinical trial, and any bias in the trial will be reflected in this study. Second, we did not have access to individual patient data from the ORIENT-11 trials, therefore, digitalization of the reported survival curves was used to replicate the survival data. Using the reconstructed survival data, a range of parametric distributions were fitted to the curves to estimate long term PFS and OS. Although several steps were taken to ensure the choice of parametric distribution for extrapolating the data is plausible, it increases the uncertainty of the model output. Third, the costs in the model did not include grade 1/2 adverse events cost, although these adverse effects were relatively minor and had a small impact on cost. Fourth, this study evaluates the economy of drugs based on the Mathematical Model Method and makes assumptions about drugs usage. However, in the real world, the clinical treatment of patients will be more diversified based on the patient's physical characteristics and disease status. Therefore, it is necessary to further evaluate the cost-effectiveness of sintilimab in the treatment of lung cancer with real-world data.

## Conclusion

After the implementation of the China new medical insurance catalogue in January 2022, sintilimab plus chemotherapy is more cost-effective compared with chemotherapy alone in China as the first-line treatment for locally advanced or metastatic squamous NSCLC patients. 

## Data Availability

The data that support the findings of this study are available from the corresponding author upon reasonable request.
