# Correction: Cost-effectiveness analysis of sintilimab plus pemetrexed and platinum versus chemotherapy alone as first-line treatment in metastatic non-squamous non–small cell lung cancer in China

**DOI:** 10.1186/s13561-023-00419-w

**Published:** 2023-01-18

**Authors:** Huiqin Liu, Ying Wang, Qi He

**Affiliations:** 1 Department of Medical Insurance, Cancer Prevention and Treatment Institute of Chengdu, Chengdu Fifth People’s Hospital (The Second Clinical Medical College, Affiliated Fifth People’s Hospital of Chengdu University of Traditional Chinese Medicine), Chengdu, China; 2 Department of Oncology, Cancer Prevention and Treatment Institute of Chengdu, Chengdu Fifth People’s Hospital (The Second Clinical Medical College, Affiliated Fifth People’s Hospital of Chengdu University of Traditional Chinese Medicine), Chengdu, China

**Correction: Health Econ Rev 12, 66 (2022)**

**https://doi.org/10.1186/s13561-022-00410-x**

Following publication of the original article [1], the authors corrected the declared corresponding author from Huiqin Liu to Qi He. Qi He was the one who put forward the idea of the article; and is the general director of the whole project.

The original article [1] has been corrected.
